# Feed‐forward loops between metastatic cancer cells and their microenvironment—the stage of escalation

**DOI:** 10.15252/emmm.202114283

**Published:** 2022-05-04

**Authors:** Zora Baumann, Priska Auf der Maur, Mohamed Bentires‐Alj

**Affiliations:** ^1^ Tumor Heterogeneity Metastasis and Resistance Department of Biomedicine University Hospital Basel University of Basel Basel Switzerland

**Keywords:** breast cancer, feed‐forward loops, interdependency, metastasis, tumor microenvironment, Cancer, Molecular Biology of Disease

## Abstract

Breast cancer is the most frequent cancer among women, and metastases in distant organs are the leading cause of the cancer‐related deaths. While survival of early‐stage breast cancer patients has increased dramatically, the 5‐year survival rate of metastatic patients has barely improved in the last 20 years. Metastases can arise up to decades after primary tumor resection, hinting at microenvironmental factors influencing the sudden outgrowth of disseminated tumor cells (DTCs). This review summarizes how the environment of the most common metastatic sites (lung, liver, bone, brain) is influenced by the primary tumor and by the varying dormancy of DTCs, with a special focus on how established metastases persist and grow in distant organs due to feed‐forward loops (FFLs). We discuss in detail the importance of FFL of cancer cells with their microenvironment including the secretome, interaction with specialized tissue‐specific cells, nutrients/metabolites, and that novel therapies should target not only the cancer cells but also the tumor microenvironment, which are thick as thieves.

## Graphical abstract

Metastases can arise decades after primary breast tumor resection. This review by M. Bentires‐Alj and colleagues describes how the environment of the most common metastatic sites is influenced by the primary tumor and the varying dormancy of disseminated tumor cells, with a focus on feed‐forward loops.

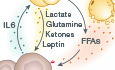

**Glossary**
Cancer dormancy: Two forms of dormancy have been observed: **Cellular dormancy** describes a reversible non‐proliferative state of a cancer cell that can last for several years. **Tumor mass dormancy** represents the offset of cancer cell proliferation by cell death, resulting in net‐constant cell numbers.
Colonization: Growth of micrometastases into macrometastases.
Disseminated tumor cells (DTCs): Cancer cells that infiltrate and survive in distant sites. DTCs may succumb, remain dormant, or colonize the tissue.
Extracellular Matrix (ECM): Three‐dimensional network surrounding cells, consisting of macromolecules and minerals, including collagens, glycoproteins, and cell adhesion proteins. The ECM provides essential structural support and serves diverse biochemical activities. Components of the ECM are produced intracellularly by resident cells and subsequently secreted into the extracellular space. The composition thus varies widely between organs and can be transiently remodeled upon physiological injury or chronic stimuli, including cancer/metastases.
Extracellular Vesicles (EV): EV is a collective term covering a variety of cell‐derived membranous structures that encapsulate and transport cellular materials, and nearly, all cell types can produce them. EV cargo include proteins, lipids, microRNAs (miRs), mRNA, and noncoding RNAs. One example of EVs are exosomes. In the context of cancer, EVs released from primary tumors can establish a pre‐metastatic niche in distant organs.
Inter‐site heterogeneity: Heterogeneity between lesions in different sites. Inter‐site heterogeneity is generally used to describe cancer cells; however, it also applies to other cell types that are part of the tumor microenvironment.
Intra‐site heterogeneity: Heterogeneity within the same primary tumor or the same metastasis. Commonly, intra‐site heterogeneity is used for cancer cells only; however, it also applies to other cell types that are part of the tumor microenvironment.
Metastasis: Metastasis is a multi‐step process in which cancer cells invade surrounding tissues at the primary site, intravasate, and survive in the circulation as circulating tumor cells (CTCs) that extravasate at distant organs (referred to as disseminated tumor cells; DTCs). Metastases are responsible for the majority of breast cancer‐related deaths.
Minimal residual disease (MRD): This disease stage is when a patient is in remission, and only a small number of cancer cells have persisted therapy. Minimal residual disease can endure for several months or, in some cases, up to decades and represents a significant challenge for long‐term remission because it is a reservoir of cancer cells that can regrow anytime. Additionally, these cells are often of a more aggressive type because they are treatment‐resistant and will therefore be more challenging to eradicate.
Organotropism: Process of cancer cells spreading to and surviving in distant organs in a non‐arbitrary way. Broadly categorized, it depends on cancer cell intrinsic factors (i.e., clonal fitness, genetic alterations, or metabolism) and non‐cancer cell autonomous features (i.e., endothelial structure, or immune cells). These factors allow seeding to specific distant organs and enable survival in this foreign microenvironment.
Pre‐metastatic Niche (PMN): Primary tumors secrete soluble factors and EVs that reprogram distant sites and facilitate future cancer cell homing and survival. Numerous factors influence PMN formation, including vascular changes, stromal cell activation, immune cell recruitment, ECM remodeling, and metabolic reprogramming.
Tumor Microenvironment (TME): The TME can include endothelial, immune, tissue‐resident cells, nerve cells, adipocytes, a stroma composed of extracellular matrix, cancer‐associated fibroblasts, mesenchymal cells, and numerous soluble factors. Breast cancer cells and the TME co‐evolve dynamically through reciprocal interactions that corrupt homeostatic networks and contribute actively to disease progression.

## Introduction

Metastases are the leading cause of cancer‐related deaths in breast cancer patients (Siegel *et al*, 2020). This spread to distant organs is a multi‐step process in which cancer cells invade surrounding tissues at the primary site, intravasate, and survive in the circulation as circulating tumor cells (CTCs) that extravasate at distant organs (referred to as disseminated tumor cells; DTCs), and possibly form metastasis (Chambers *et al*, 2002; Alix‐Panabières & Pantel, 2014; Lambert *et al*, 2017; Esposito *et al*, 2021; Ganesh & Massagué, 2021). Especially, CTC clusters are rare but are more metastatic than single CTCs (Aceto *et al*, 2014). Despite significant advances in our understanding of the metastatic cascade, therapeutic targeting of metastasis remains poor and is a significant impediment to the clinical management of patients. Much of the reduction in cancer‐related mortality achieved by therapeutic interventions has been based on early detection and improved surgery, combined with adjuvant treatments that eliminate DTCs. However, for breast cancer patients that reach the metastatic stage, the 5‐year survival rate has barely improved in the last 20 years (Esposito *et al*, 2021). Therefore, a mechanistic understanding of the metastatic odyssey that suggests specific effective therapies is of the utmost clinical importance.

One of the main challenges for successful treatment is tumor heterogeneity, which is found at several levels within a single patient. First, there is the heterogeneity within the same primary tumor or the same metastases, known as intra‐site heterogeneity. Second, there is heterogeneity between different lesions, so‐called inter‐site heterogeneity (Koren & Bentires‐Alj, 2015; Lüönd *et al*, 2021). Over the years, several studies have shed light on the diverse origins of genetic heterogeneity during cancer evolution (Burrell *et al*, 2013; Yates, 2017). While metastases of breast cancer patients harbor more mutations than primary tumors (Angus *et al*, 2019; Priestley *et al*, 2019), the gain of actionable driver mutations seems to play a subordinate role in the metastatic process (Vanharanta & Massagué, 2013; van de Haar *et al*, 2021; Reiter *et al*, 2018). Therefore, phenotypic alterations of DTCs, including changes in epigenome, metabolism, and interactions with immune and stromal cells, seem to be key for metastatic progression. The combination of genetic heterogeneity, phenotypic plasticity, and various selection pressures at different stages of the metastatic progression is a major hurdle to successful therapy.

Cancers can arise from a single “mutated” cell; however, disease progression is often a consequence of sequential alterations that enrich for aggressive subpopulations within the tumor (Swanton, 2012). To a certain degree, cancer progression resembles the Darwinian “survival of the fittest” principle and tumors can be viewed as constantly evolving ecosystems (Tabassum & Polyak, 2015; Vendramin *et al*, 2021). One of the most important non‐genetic drivers of cancer development is the tumor microenvironment (McGranahan & Swanton, 2017; Black & McGranahan, 2021). Tumor development is heavily shaped by physical and architectural constraints of the tissue, competition for space, the enduring effects of the immune system, and the changing nutritional environment (Altea‐Manzano *et al*, 2020). In particular, the reactions of cancer cells to the potentially harsh environment outside of primary tumors is a driver in the selection of metastatic cancer clones (Massagué & Obenauf, 2016; Vendramin *et al*, 2021).

This review summarizes how the environment of metastatic sites is influenced by the phenotype of the primary tumor and by the varying dormancy of DTCs, with a special focus on how established metastases persist and grow in distant organs due to feed‐forward loops (FFLs). Finally, we discuss the concept that novel therapies should target not only the cancer cells but also the tumor microenvironment.

## The (co‐)evolution of metastasis and the microenvironment

### Properties of the pre‐metastatic niche

An elaborate interplay of the primary tumor’s secretome with immune and tissue‐resident cells results in a microenvironment in secondary organs (the pre‐metastatic niche—PMN) that favors subsequent cancer cell homing. PMN alterations can be broadly categorized into (i) vascular changes including vascular leakiness, expression of adhesion molecules, clot formation, (ii) activation of stromal components and extracellular matrix (ECM) reorganization, for example, by distant secretion of matrix metalloproteinases, (iii) immune cell recruitment, (iv) changes in resident cells, including metabolic adaptions (Peinado *et al*, 2017; Wang *et al*, 2019).

The concept that metastases do not seed arbitrarily was proposed in 1889 by Stephen Paget, who suggested that cancer cells (“seeds”) preferentially home to specific secondary organs (“soil”) (Paget, 1889). It is nowadays evident that the “seeds” can prime the “soil” on multiple levels. The “seed and soil” theory was amended with the concept of the PMN. Systemic effects of tumor‐secreted factors and vesicles result in changes in secondary organs devoid of cancer cells (the PMN) that favor subsequent CTC homing (Peinado *et al*, 2017).

Organ‐specific metastases (organotropism) are a common occurrence in multiple solid cancers. Clinically, breast cancer often spreads to several distant organs and different subtypes have been associated with differential patterns of metastasis. The most common site of metastasis shared between all subtypes is the bone, with the highest percentage in the estrogen receptor (ER)‐positive subtype. In ER‐positive/HER2‐negative patients, there is a pattern of bone only disease (Leone *et al*, 2017). Patients with triple‐negative breast cancer have a higher risk to develop brain and lung metastases than patients with other subtypes. On the other hand, HER2‐positive tumors more often spread to the liver (Chen *et al*, 2017; Wu *et al*, 2017). Moreover, there is a 25‐50% incidence of brain metastasis in advanced HER2‐positive disease (Zimmer *et al*, 2020).

Multiple factors contribute to organotropism; for example, secretion of lysyl oxidase by the hypoxic primary tumor induces Wnt signaling in pre‐metastatic bone lesions (Cox *et al*, 2015); periostin expression in resident cells modifies the ECM and enhances lung metastases (Malanchi *et al*, 2012); and lactate secretion by cancer cells limits NK cell cytotoxicity and increases brain metastases (Parida *et al*, 2022).

### The metastatic niche

**Figure 1:**
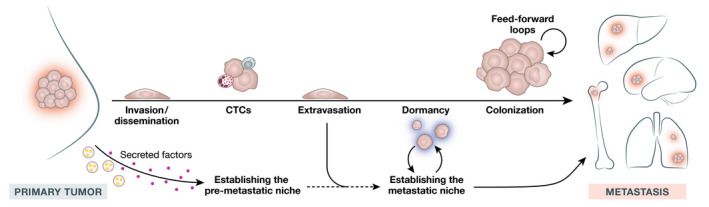
The path of cancer escalation Breast cancer disease progresses over several stages. After local invasion and dissemination from the primary tumor, CTCs enter the blood circulation, traveling as single cells or in clusters combined with neutrophils or T cells. Several factors secreted from primary tumor cells, including exosomes, S100 proteins, VEGF‐A, TGFβ, TNFα, SAAs, and CCL2, influence the properties of pre‐metastatic niches in particular organs). Additionally, these same factors can facilitate the extravasation of DTCs at distant sites. Metastatic cancer cells adhere to and multiply at distant sites (pre‐metastatic niches) with surface and immunological affinities. Cancer cell dormancy can, at this stage, persist for several years or even decades. Disseminated cancer cells are in constant interaction with tissue‐resident cells and with the resident homeostatic programs that foster metastatic niche formation and survival. This results in self‐enhancing loops and disease escalation that is in many cases fatal. CTCs, circulating tumor cells; CCL2, C‐C motif chemokine ligand 2; DTCs, disseminated tumor cells; SAAs, serum amyloid A proteins; TGFβ, transforming growth factor‐beta; TNFα, tumor necrosis factor‐alpha; VEGF‐A, vascular endothelial growth factor‐A.

After extravasation into the blood stream (Fig 1), only approximately 0.01% of CTCs infiltrate and eventually colonize distant organs (Massagué & Obenauf, 2016). The initial arrest of CTCs at distant sites is regulated primarily by blood circulation and flow patterns, the vascular architecture, and whether they adhere to the endothelium (Chambers *et al*, 2002). CTC survival is promoted by neutrophils in the circulation and the PMN (Szczerba *et al*, 2019). Additionally, CTC homing to distant sites is enhanced by neutrophils and macrophages facilitate anchorage and escape from immune surveillance and provide survival signals and metabolites (Kitamura *et al*, 2015; Celià‐Terrassa & Kang, 2018). Once DTCs intravasate into distant organs, whether they succumb, remain dormant, or colonize the tissue depends on their surroundings (Fig 1) (Lukanidin & Sleeman, 2012; Liu & Cao, 2016). In patients, metastasis cannot be fully distinguished from therapy resistance as most metastases of breast cancer are recurrences after systemic therapy (Weiss *et al*, 2022). Thus, the metastatic niche is not only important for metastatic growth but also for resistance to therapy.

### Dormancy

Following primary tumor removal and therapy, minimal residual disease (MRD) that persists several years to decades without clinical detection is referred to as “dormancy”. Two forms of dormancy have been proposed: cellular dormancy and tumor mass dormancy. Cellular dormancy is characterized by three traits: dormant DTCs persist in foreign organs (“soil”), they are—for the time being—arrested in G_0_ and are frequently resistant to treatments (Ghajar, 2015). In tumor mass dormancy, cancer cell proliferation is offset by cell death due to immune surveillance and/or insufficient vascularization, resulting in insignificant net change in cell number (Chambers *et al*, 2002; Kang & Pantel, 2013). Notably, the risk of breast cancer relapse is subtype‐dependent. While this risk remains constant in the ER‐positive subtype, it decreases over time in ER‐negative disease (Lee & Djamgoz, 2018; Rueda *et al*, 2019).

Cancer cells may enter or exit cellular dormancy through cancer cell‐intrinsic mechanisms (Vera‐Ramirez *et al*, 2018; La Belle Flynn *et al*, 2019) or extrinsic stimuli (Ghajar *et al*, 2013; Senft & Jeremias, 2019; Perego *et al*, 2020; Correia *et al*, 2021). We have shown recently that IFNγ secretion from NK cells maintains breast cancer cell dormancy in the liver (Correia *et al*, 2021). The reawakening of DTCs and subsequent colonization is mediated by CXCL12 secretion from activated hepatic stellate cells. This suppresses immune surveillance by inducing NK cell quiescence and results in metastatic outgrowth (Correia *et al*, 2021; Lopes & Vivier, 2021). Additionally, it was reported that tissue‐resident type I innate lymphoid cells promote metastatic seeding to the liver, whereas conventional NK cells impede colonization (Ducimetière *et al*, 2021).

Other mechanisms have been reported to tip the balance from dormancy to overt metastasis. Neutrophil activity can wake dormant DTCs upon exposure to environmental inflammatory cues (Albrengues *et al*, 2018). Depending on the host’s NK cell status, neutrophils can be facilitators or inhibitors of metastatic colonization (Li *et al*, 2020). Additionally, sprouting neovasculature can lead to breast cancer DTC outgrowth (Ghajar *et al*, 2013). Moreover, surgical resection of primary breast tumors can also trigger outgrowth of previously immune‐controlled metastases by macrophage engagement (Krall *et al*, 2018). Unfortunately, the triggers leading to clinically detectable metastases are probably the least characterized—yet potentially most important components—in the metastatic cascade (Esposito *et al*, 2018). Admittedly, monitoring and studying the switch from dormancy to metastatic outgrowth in clinical specimens is challenging and most of our current understanding of these processes is derived from preclinical studies.

### Colonization and feed‐forward loops (FFLs)—the stage of escalation

Colonization (the transition from a micro to macro metastasis) is the last and most fatal stage of breast cancer and the most difficult to treat due to tumor heterogeneity, metabolic flexibility, and complex interactions of cancer cells with the tumor microenvironment. It is important to note that no single therapeutic agent has been approved that specifically targets breast cancer metastases. That clinically detectable metastases can develop years after resection of the primary tumor highlights the fact that colonization is the most complex and rate‐limiting phase of the metastatic cascade (Fig 1) (Massagué & Obenauf, 2016). The signals governing the development of nascent metastatic lesions are still being defined, but it is already known that stimuli range from stress hormones to chemokines (Obradović *et al*, 2019; Ozga *et al*, 2021).

One example is CCL2 secretion from mammary tumors, which recruits C‐C motif receptor 2 (CCR2)‐expressing inflammatory monocytes to the metastatic site (Qian *et al*, 2011; Bonapace *et al*, 2014; Kitamura *et al*, 2015). We have shown previously that anti‐CCL2 therapy efficiently retains monocytes in the bone marrow. However, interruption of the treatment results in rapid dissemination and direct colonization of metastatic cells fueled by elevated monocyte release from the bone marrow due to vascular endothelial growth factor A (VEGF‐A) and interleukin 6 (IL6) signaling (Bonapace *et al*, 2014). This highlights that targeting colonization is complicated by interactions between tumor cells, their microenvironment, and remote sites such as the bone marrow (Keklikoglou & De Palma, 2014; Hitchcock & Watson, 2015).

The outgrowth of metastases can be influenced by factors from resident immune and stromal cells. However, crosstalk between cancer cells and the tumor microenvironment (TME) can also result in detrimental self‐enhancing loops that increase the survival and proliferation of metastatic tumor cells. One example is that mammary tumor‐initiating cells exhibit elevated G‐CSF production due to increased mTOR signaling, which then leads to accumulation of myeloid‐derived suppressor cells (MDSC). Notch activation by MDSCs subsequently increase tumor‐cell initiating frequency (Welte *et al*, 2016). Such vicious cycles vary between different tissues and are therefore discussed separately for specific organs later in this review. These organ‐specific self‐enhancing loops are challenging but potentially offer important therapeutic opportunities for treatment of metastases that will be discussed at the end of this review.

## Factors influencing the TME

### The secretome of cancer cells and their microenvironment

**Figure 2:**
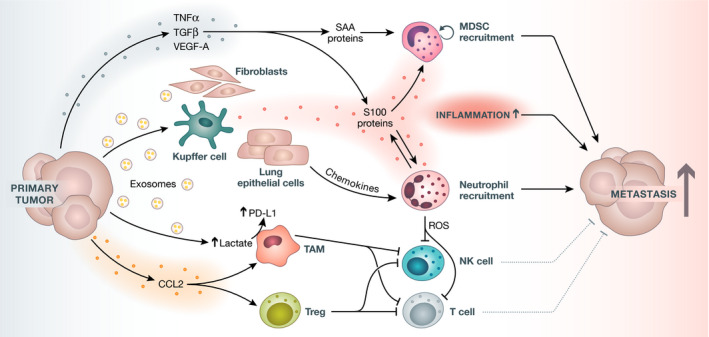
The intricate soluble network that creates a (pre‐)metastatic niche The vast secretome of primary tumors includes chemokines, growth factors, and a range of tumor vesicles, including exosomes. These enter the blood system and reach secondary sites before cancer cells disseminate. This results in a microenvironment that is compatible for tumor cells and immune‐suppressive. Examples include TNFα, TGFβ, and VEGF‐A, which induce serum amyloid A (SAA) proteins that recruit MDSCs to the organ. Additionally, S100 proteins are induced and secreted by various mechanisms, including chemokines and exosomes, from the primary tumor that act on tissue‐resident cells, including fibroblasts, Kupffer cells, and lung epithelial cells. S100 proteins are pro‐inflammatory proteins diversely involved in metastatic niche properties. Furthermore, S100 proteins secreted from immune‐suppressive immune cells including MDSCs are additionally able to initiate their proliferation in an S100‐autocrine manner. The proteins are also involved in a vicious FFL with neutrophils that are attracted to the niche, where they in turn secrete even more S100 proteins. Exosomes induce higher lactate secretion from tumor‐associated macrophages (TAM), which in turn upregulates PD‐L1, a crucial immune checkpoint inhibitor. Moreover, CCL2 from primary tumors attracts TAMs and regulatory T cells (Tregs) to pre‐metastatic niches, which deters anti‐tumorigenic NK cells and T cells. In addition, reactive oxygen species (ROS) secreted from neutrophils further impact cytotoxic NK cells and T cells. All of these listed factors promote metastatic signaling and thus enhance exacerbating disease progression. CCL2, C‐C motif chemokine ligand 2; MDSC, myeloid‐derived suppressor cells; PD‐L1, programmed death‐ligand 1; ROS, reactive oxygen species; SAA, serum amyloid A proteins; TAM, tumor‐associated macrophages; TGFβ, transforming growth factor β; TNFα, tumor necrosis factor α; Treg, regulatory T cells; VEGF‐A, vascular endothelial growth factor‐A.

A key aspect of metastasis initiation is the cancer secretome, that is, cytokines, metabolites, and extracellular vesicles that transmit reprogramming signals to cells in the vicinity of the cancer as well as in distant organs. The secretome is relevant from the onset of tumorigenesis and embodies crucial stimuli throughout the metastatic cascade. In the following paragraph, we focus on initial FFLs that prime and establish the metastatic niche (Fig 2).

The PMN is composed of several soluble factors secreted by cancer cells in the primary tumor, by bone marrow‐derived cells, suppressive immune cells, and host tissue stromal cells (Paolillo & Schinelli, 2019). The main pro‐inflammatory signal proteins secreted from the cancer cells include VEGF‐A, transforming growth factor β (TGFβ), and tumor necrosis factor (TNF) (Paolillo & Schinelli, 2019). In turn, all these factors further induce expression of S100 chemoattractants, a family of Ca2^+^‐binding proteins that are implicated in many aspects of cancer progression (Lukanidin & Sleeman, 2012; Rinaldi *et al*, 2021). S100 proteins lie at the center of many vicious FFLs that foster metastasis. Examples include S100A8 and S100A9 secreted from breast cancer cells, as well as MDSCs that promote metastases in xenograft models in an auto‐ and paracrine fashion (Bresnick *et al*, 2015).

The cancer secretome is critical for recruitment of immune cells. Tumor‐derived cytokines and chemokines such as CCL2, mentioned above, recruit regulatory and immunosuppressive immune cells, including tumor‐associated macrophages (TAMs), to secondary organs, where they are potent orchestrators of PMN formation through immune‐modulation and immune‐suppression (Qian *et al*, 2011; Bonapace *et al*, 2014; Ozga *et al*, 2021). Recently, β‐catenin‐mediated CCL2 secretion has been implicated in enhancing TAM recruitment and promoting metastasis (Zhang *et al*, 2021a). For an extensive overview of chemokines and the immune response to cancer, we refer to the recent review by Ozga *et al* (2021). Importantly, the same cytokines are often involved in recruiting both pro‐ and anti‐tumorigenic immune cells. For example, the CCR5/CCL5 axis attracts both Tregs and cytotoxic CD8^+^ T cells (Ozga *et al*, 2021). Ultimately, cytokines and chemokines are double‐edged swords as they are indispensable for homeostatic tissue functions but may become delinquent in metastases formation.

Heterogeneous immunosuppressive bone marrow‐derived cells recruited by the cancer secretome are also important contributors to the PMN. Examples include the ability of VEGF to mobilize VEGFR1^+^ hematopoietic bone marrow progenitor (HPCs) cells or VEGF‐A, TGFβ, and TNFα‐evoked induction of serum amyloid A proteins that recruit MDSCs (Kaplan *et al*, 2005; Hiratsuka *et al*, 2006, 2008). MDSCs are a heterogenous population of immunosuppressive cells that can be broadly categorized into CD11b^+^CD68^+^F4/80^+^ myeloid cells, CD11b^+^Ly6C^+^ monocytes, and CD11b^+^Ly6G^+^Ly6C^+^ granulocytes (Liu & Cao, 2016). MDSCs expression of integrins, and secretion of chemokines, inflammatory mediators, and growth and angiogenic factors promote the PMN (Liu & Cao, 2016). Intriguingly, MDSCs can reduce NK cell cytotoxicity in the pre‐metastatic lung in breast cancer models (Sceneay *et al*, 2012). For further reading on MDSCs, we refer to a review by Wang *et al* (2019).

Neutrophils are another important immune cell type that is attracted by tumor‐derived factors, including G‐CSF and S100 proteins, as well as by the CXCL12/CXCR4 axis (Dumitru *et al*, 2013; Leach *et al*, 2019; Gonzalez *et al*, 2020; Wang *et al*, 2020). Leukotriene signaling from neutrophils can support metastasis‐initiating cells in pre‐metastatic lung of mammary cancer mouse models (Wculek & Malanchi, 2015). This also contributes to the reawakening of dormant tumor cells by neutrophil extracellular traps (NETs) formation (Albrengues *et al*, 2018) and to metastatic colonization, as they are recruited by γδ‐T cell‐released G‐CSF that leads to expansion and polarization of neutrophils in the lung metastatic niche (Coffelt *et al*, 2015). Moreover primary tumor angiopoietin‐like protein 2 (ANGPTL2) secretion increases recruitment of neutrophils to the lung, thus contributing to PMN formation (Charan *et al*, 2020).

Extracellular vesicles (EVs), including exosomes released from the primary tumor, can dictate organotropic behavior of metastatic tumor cells (Hoshino *et al*, 2015). Breast cancer‐derived exosomes containing several integrins travel in the bloodstream and are preferentially endocytosed into organ‐specific cells such as lung fibroblasts or liver Kupffer cells (Hoshino *et al*, 2015). Various S100 family proteins in these cells are then highly upregulated and secreted, leading to a pro‐inflammatory supportive niche (Sakaguchi, 2017). Additionally, exosome‐packaged RNA can activate Toll‐like receptor 3 (TLR3) signaling in lung epithelial cells, leading to chemokine secretion and subsequent neutrophil attraction to the PMN (Liu *et al*, 2016). Tumor‐derived exosomes were shown to render macrophages in the PMN immunosuppressive through glycolytic dominant metabolic reprogramming (Morrissey *et al*, 2021). Mechanistically, NF‐κB augments glycolysis, which increases lactate and drives PD‐L1 expression (Morrissey *et al*, 2021).

Additionally, EVs can contain microRNAs (miRs), small non‐coding RNAs that are crucial master regulators of gene expression in several cancer‐related signaling pathways (Chen *et al*, 2018). One example is miR122, which reprograms glucose metabolism and promotes breast cancer metastasis in preclinical and clinical studies (Wu *et al*, 2012; Fong *et al*, 2015). Another example is miR‐105, which destroys endothelial barriers and promotes metastasis (Zhou *et al*, 2014). Furthermore, brain metastatic cells release miR‐181c‐containing EVs capable of destructing the blood–brain barrier (Tominaga *et al*, 2015).

Early detection of metastasis is an unmet clinical need. Recently, a study of 426 human samples of multiple cancers (including breast cancer) identified EV markers from tumor and plasma that might improve early cancer detection and characterization (Hoshino *et al*, 2020). Additionally, cancer exosomal migration‐inducing and hyaluronan‐binding protein (CEMIP) may be a potential prognostic factor for brain metastasis (Rodrigues *et al*, 2019). For further reading regarding how exosomes promote metastasis, we refer to the review by Wortzel *et al* (2019).

While several studies have shown that tumor‐secreted vesicles can modulate distant organs in various ways, thereby creating an intricate regulatory network, we are only at the beginning of uncovering the multiplicity of effects of EVs in metastasis. In summary, the secretome of cancer cells and the TME are potent and multifaceted modulators that can enhance disease progression and, thus, warrant careful investigation.

## Interaction with specialized tissue‐resident cells

### Lung

The lung is exposed to the external environment and exhibits various efficient mechanisms that eliminate pathogens, primarily innate immune responses. Inflammation and immune responses in the lung are limited and, thus, tissue damage is avoided. However, this characteristic is a drawback that exposes the lungs to DTCs outgrowth (Gao *et al*, 2019).

**Figure 3:**
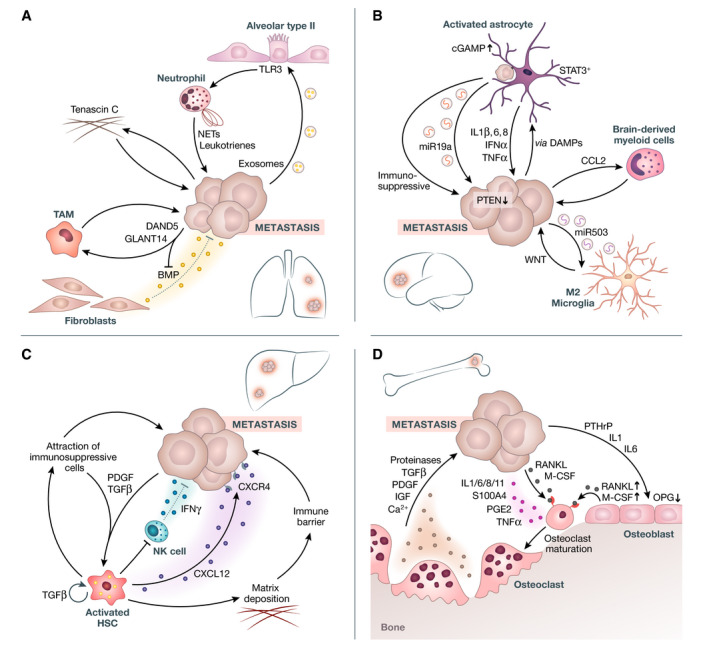
Interaction with tissue‐resident cells and how these interactions facilitate colonization During colonization, cancer cells coalesce with tissue‐resident cells, which results in vicious cycles of cancer maintenance and nurturing. (A) In the lung, exosomal RNAs secreted from metastatic cells activate TLR3 in alveolar type II cells and induce recruitment of neutrophils. The secretion of leukotrienes and the expulsion of NETs from neutrophils can enhance cancer metastasis. Tenascin C from tumor cells facilitates colonization through WNT and Notch signaling in cancer cells. DAND5 and GLANT14 secreted from cancer cells recruit TAMs and further enhance metastatic colonization. Furthermore, GLANT14 blocks inhibitory BMPs secreted from tissue‐resident fibroblasts. (B) In the brain, DAMPs from DTCs can activate resident astrocytes and miR19a exosomes, IL1β IL6, IL8, IFNα, and TNFα secreted from these activated astrocytes enhance metastatic growth. A STAT3+ subpopulation of astrocytes has immunosuppressive properties. CCL2 secreted from tumor cells attracts pro‐tumorigenic brain‐derived myeloid cells. Tumor‐derived exosomes with miR503 can induce M2‐polarization of microglia, which enhances metastatic growth through WNT signaling. (C) TGFβ and PDGF are secreted from DTCs in the liver and activate HSCs. aHSCs‐secreted CXCL12 evoke NK cell quiescence via its cognate receptor CXCR4, and thus prevent them from retaining DTCs in a dormant state. Additionally, CXCL12 secreted from aHSCs binds to the CXCR4 of cancer cells and enhances their growth. The deposition of matrix from aHSCs forms a barrier against immune cells. Furthermore, aHSCs attract immunosuppressive immune cells, from which further TGFβ and PDGF is secreted and thus even more HSCs are activated. (D) The bone is a classic example of tumor‐cell occupation of the microenvironment of a distant site. Several factors (IL1, IL6, IL8, IL11, S100A4, PGE2, TNFα, RANKL, M‐CSF) secreted from DTCs induce pre‐osteoclast maturation to osteoclasts. PTHrP, IL1, and IL6 secreted from DTCs enhance RANKL and M‐CSF and downregulate OPG expression in osteoblasts, thus further inducing the maturation of osteoblasts. Dysbalance of osteoclast/osteoblasts leads to bone resorption and liberation of pro‐metastatic nutrients, enzymes, and growth factors (Ca2+, IGF, PDGF, TGFβ, and proteinases) from the bone microenvironment. BMPs, Bone morphogenetic proteins; CCL2, C‐C motif chemokine ligand 2; CXCL12, C‐X‐C motif chemokine ligand 12; CXCR4, C‐X‐C motif chemokine receptor 4; DAMPs, damage‐associated molecular patterns; DAND5, DAN domain BMP antagonist family member 5; DTCs, disseminated tumor cells; GLANT14, polypeptide N‐acetylgalactosaminyltransferase 14; (a)HSCs, (activated) hepatic stellate cells; IFG, insulin‐like growth factor; IL, interleukin; M‐CSF, macrophage colony‐stimulating factor; miR, micro RNA; NETs, neutrophil extracellular traps; OPG, osteoprotegerin; PDGF, platelet‐derived growth factor; PGE2, prostaglandin E2; PTHrP, parathyroid hormone‐related protein; RANKL, receptor activator of nuclear factor kappa B ligand; STAT3, signal transducer and activator of transcription 3; TGFβ, transforming growth factor‐beta; TNFα, tumor necrosis factor‐alpha; TLR3, toll‐like receptor 3; VEGF‐A, vascular endothelial growth factor‐A.

Secretion of bone morphogenic factors (BMPs) from lung‐resident fibroblasts is inhibitory for metastatic cancer cells (Fig 3A). However, this effect is cancelled by the activity of DAN domain family member 5 (DAND5 or COCO), a secreted antagonist of TGFβ ligands, and N‐acetyl‐galactosaminyltransferases 14 (GALNT14), which inhibit BMPs. The result is reactivation of dormant DTCs and subsequent metastatic outgrowth (Song *et al*, 2016). Cancer‐secreted GALNTs can also attract macrophages to the metastatic site, further promoting the growth of metastatic cancer cells in the lung through fibroblast growth factor (FGF) secretion (Song *et al*, 2016).

The ECM protein tenascin C (TNC) secreted from breast cancer cells arriving in the lung initiates the formation of a metastatic micro‐niche (Fig 3A) (Oskarsson *et al*, 2011). TNC promotes the outgrowth and survival of pulmonary metastases, allowing cancer cell survival before the metastatic niche is supported by the surrounding lung stroma (Gao *et al*, 2019).

Tumor exosomal RNAs activate TLR3 signaling in alveolar type II epithelial cells, which recruits neutrophils and contributes to the formation of a PMN (Fig 3A) (Liu *et al*, 2016; Altorki *et al*, 2019). Additionally, it was reported that neutrophil activity can reawaken dormant DTCs through neutrophil extracellular traps (NETs)‐induced inflammation and subsequent integrin α3β1 signaling‐dependent proliferation of dormant DTCs (Albrengues *et al*, 2018). Tumor‐secreted protease cathepsin C (CTSC) activity also enhances breast cancer metastasis through recruitment of neutrophils and formation of NETs (Xiao *et al*, 2021). Leukotrienes secreted from neutrophils can directly promote lung colonization, which results in selective expansion of highly tumorigenic cancer cells (Wculek & Malanchi, 2015). To summarize, different mechanisms involving neutrophils unshackle DTCs in the lung by generating FFLs with cancer cells and alveolar type II epithelial cells.

### Brain

In the brain microenvironment, interaction between DTCs and reactive astrocytes or microglia sparks several vicious cycles that promote metastasis formation, driving disease progression, and immune evasion.

DTCs that reach the brain can activate astrocytes in the surrounding tissue, likely through damage‐associated molecular pattern (DAMP) signals (Fig 3B). Initially, the number of DTCs is restricted by these reactive astrocytes, primarily through plasmin activation. However, serpins secreted by cancer cells block the proteolytic activation of plasmin and enhance DTC accumulation (Valiente *et al*, 2018). Cancer cells established in the brain microenvironment are influenced by the pro‐tumorigenic activity of reactive astrocytes. Gap junctions that arise between metastatic cells and reactive astrocytes induce cyclic monophosphate‐adenosine monophosphate (cGAMP)‐mediated paracrine loops (Fig 3B) (Srinivasan *et al*, 2021). This and further mechanisms result in the release of multiple inflammatory cytokines from reactive astrocytes, including IL1β, IL6, IL8, IFNα, or TNFα, which then drive the survival and colonization of cancer cells, as well as chemo‐resistance through calcium sequestration (Lin *et al*, 2010; Kim *et al*, 2011; Xing *et al*, 2013; Chen *et al*, 2016, 2018; Valiente *et al*, 2018).

In addition to inflammatory cytokines, miR19a‐containing exosomes are released from reactive astrocytes and reduce expression of the tumor suppressor PTEN in metastatic cells (Fig 3B). Increased secretion of the chemokine CCL2 from DTCs then attracts pro‐tumorigenic brain‐derived myeloid cells to the metastatic site (Zhang *et al*, 2015). Reactive astrocytes also have immunosuppressive effects through inhibition of CD8^+^ T cells and polarization of TAMs into anti‐inflammatory M2 macrophages in a STAT3‐dependent manner (Fig 3B) (Priego *et al*, 2018; McFarland & Benveniste, 2019). STAT3 labels a subpopulation of reactive astrocytes found near metastatic lesions that have been shown to be important for establishing a pro‐metastatic environment (Priego *et al*, 2018). Another FFL stimulated by brain metastases involves increased expression of hepatocyte growth factor (HGF) receptor (c‐Met) in tumor cells that leads to high IL1β secretion (Fig 3B). This results in increased HGF secretion from tumor‐associated reactive astrocytes, which feeds back to the c‐Met receptor. HGF‐dependent c‐Met‐IL1β signaling also enhances the secretion of pro‐tumorigenic IL8 and CXCL1, which promotes angiogenesis (Xing *et al*, 2016).

Microglia are tissue‐resident macrophages in the brain closely interacting with DTCs. They have anti‐ and pro‐metastatic properties similar to other macrophage populations. In breast cancer cells, loss of the long non‐coding RNA X‐inactive–specific transcript (XIST) increases the secretion of exosomes carrying miR503 (Fig 3B). Exosomal miR503 induces M2 polarization of microglia, which then increases the colonization of breast cancer cells in the brain in a WNT‐dependent manner (Pukrop *et al*, 2010; Xing *et al*, 2018).

### Liver

The liver is one of the organs most affected by breast cancer metastasis, which often results in fatal disease progression (Harbeck *et al*, 2019). Important for this process are hepatic stellate cells (HSCs), a liver‐resident cell type. HSCs can be activated by different factors, including the fibrogenic factors TGFβ and PDGF (Tsuchida & Friedman, 2017). These and other inflammatory signals are secreted from tissue‐resident fibroblasts or macrophages upon tissue damage and may also be secreted from DTCs themselves (Fig 3C) (Tsuchida & Friedman, 2017). Secretion of CXCL12 from activated HSCs enhances metastatic colonization by binding to CXCR4 on cancer cells and thereby promoting survival and proliferation (Luker & Luker, 2006; Shi *et al*, 2020; Zielińska & Katanaev, 2020). Additionally, enhanced extracellular matrix deposition from activated HSCs induces a fibrotic liver environment that enhances metastatic growth (Fig 3C) (Marvin *et al*, 2020). This fibrosis is a physical barrier to anti‐tumorigenic immune cells (Tsuchida & Friedman, 2017). Pro‐tumorigenic immune cells associated with activated HSCs shift the balance increasingly toward a pro‐metastatic immune environment (Fig 3C). Ultimately, enhanced colonization and growth of metastatic cancer cells in the liver leads to further augmented secretion of TGFβ from the metastatic cells, which nurtures this vicious FFL (Kang *et al*, 2011). Additionally, in this loop, liver tissue‐resident NK‐cells are suppressed by TGFβ or CXCL12 secretion (Bellone *et al*, 1995; Viel *et al*, 2016; Correia *et al*, 2021). Interestingly, we have shown recently that IFNy secreted from NK cells in the liver maintains DTCs in a dormant state (Fig 3C) (Correia *et al*, 2021). Notably, activation of HSCs by chemical liver damage impairs NK cells (Correia *et al*, 2021). When dormancy is overcome, cancer cells are promoted by the same FFL described above. This highlights how the activity of HSCs results in a permissive environment for cancer cells that invade the liver.

### Bone

Bones are the most common site of metastases in breast cancer patients (Esposito *et al*, 2018). In the bone microenvironment, cancer cells interact with osteoblasts and osteoclasts cells in the hematopoietic niche. Metastatic breast cancer cells in this microenvironment often show markers of bone cells, an adaptation called osteomimicry (Rucci & Teti, 2010; Weilbaecher *et al*, 2011). Metastatic cancer cell activity upregulates osteoblast‐specific markers, including alkaline phosphatase (ALP) and Runt‐related transcription factor (RUNX2), as well as factors regulating bone turnover, such as osteoprotegerin, osteopontin, parathyroid hormone‐related peptide (PTHrP), receptor activator of nuclear factor kappa‐B ligand (RANKL), and macrophage colony‐stimulating factor (M‐CSF) (Fig 3D) (Gao *et al*, 2019). Osteomimicry can be influenced by miR218, which directly regulates osteomimetic genes and increases Wnt signaling (Gao *et al*, 2019). This adaptation promotes maturation of osteoclasts independently of osteoblast activity (Anborgh *et al*, 2010). Moreover, tumor‐derived factors, such as IL1, IL6, IL8, IL11, TNFα, prostaglandin E2 (PGE2), and S100A4 protein activate osteoclasts in a RANKL‐dependent or independent manner (Fig 3D) (Kim *et al*, 2019; Haider *et al*, 2021; Venetis *et al*, 2021).

An elevated ratio of osteoclast versus osteoblast results in bone resorption that not only liberates excessive nutrients such as calcium, serine, glycine, glucose, and glycerol but also increases growth factors, proteinases, and cytokines, including TGFβ, IGF, and PDGF (Fig 3D) (Lynch, 2011; Shi *et al*, 2014). Nutrients and growth factors released from the bone matrix have a strong metastasis‐promoting effect (Kingsley *et al*, 2007). The presence of cancer cells thereby results in a vicious cycle of osteoblast activity and osteoclastogenesis that feeds back and enhances tumor growth (Esposito *et al*, 2018). Notably, it has been shown recently that the bone microenvironment enhances phenotypic EZH2‐mediated plasticity of ER‐positive DTCs, which boosts further dissemination of cancer cells to other organs (Bado *et al*, 2021). The bone microenvironment can also enhance the metastatic spread in triple‐negative breast cancer models (Zhang *et al*, 2021b).

Osteoclast‐mediated bone resorption can also modulate the hematopoietic niche and elicit proliferation of immature bone marrow progenitors (Kollet *et al*, 2006). Furthermore, interaction of metastatic cancer cells with the hematopoietic niche is mediated by α4β1‐vascular cell adhesion molecule 1 (VCAM1), chemokines like CXCL12, BMP, Notch, Nestin, and osteopontin (Weilbaecher *et al*, 2011). Most of these proteins/pathways are also involved in the influx of metastatic cancer cells into the bone and can directly influence cancer cell proliferation and resistance to therapy.

## Fibroblasts—engineers of the metastatic niche

Fibroblasts make up a major fraction of stromal cells in the tumor environment of primary and secondary sites and contribute to several, if not all, hallmarks of cancer (Houthuijzen & Jonkers, 2018). However, other studies suggest that fibroblasts can be tumor suppressive, especially when they constitute a restrictive barrier in early tumor stages impeding invasion of tumor cells (Xu *et al*, 2010; Chang *et al*, 2012; Costa *et al*, 2018; Ping *et al*, 2021). Recent technological advances have shed more light on the heterogeneity of fibroblasts in patient samples (Costa *et al*, 2018; Pelon *et al*, 2020). They have important homeostatic activity in various organs. Fibroblasts become activated following tissue damage and produce TGFβ, which is a vital factor in wound healing (Gabbiani, 2003). Activated fibroblasts (= myofibroblasts) express alpha‐smooth muscle actin (αSMA), which is associated with a highly contractile phenotype (Rockey *et al*, 2013). Among these, are cancer‐associated fibroblasts (CAFs) that arise from tissue‐resident fibroblasts or stellate cells (in the liver and pancreas) upon exposure to cancer‐associated stimuli. CAFs can also develop from bone marrow‐derived mesenchymal stem cells that are attracted to the tumor or through differentiation of endothelial cells, adipocytes, or pericytes. Typically, CAFs are defined by their elongated and spindle‐like morphology, the lack of lineage markers for epithelial cells, endothelial cells, and leukocytes, and the absence of mutations found in cancer cells (Sahai *et al*, 2020). CAFs express mesenchymal markers such as vimentin, αSMA, fibroblast activation protein (FAP), fibroblast‐specific protein 1 (FSP1 or S100A4), and platelet‐derived growth factor‐alpha (PDGFα) (Sahai *et al*, 2020; Ping *et al*, 2021). CAFs can have different effects on the TME. Here, we will focus on mechanisms leading to positive feedback‐loops that result in disease progression and metastasis.

TGFβ1 is a well‐described stimulus that converts fibroblasts to myofibroblasts (or CAFs) (Petersen *et al*, 2003; Karagiannis *et al*, 2012). Interestingly, CAFs produce TGFβ1 upon activation, which sustains an autocrine loop of persistent CAF activation (Kakarla *et al*, 2012; Zarzynska, 2014). In CAFs, TGFβ1 elevates fibronectin, αSMA and laminin expression, which augments CAF proliferation and breast cancer cell migration (Houthuijzen & Jonkers, 2018). Likewise, it has been shown that integrin α6β4‐ and α6β1‐positive exosomes secreted from breast cancer cells can enhance expression of the pro‐inflammatory S100A4 protein in lung‐resident fibroblasts. This leads to a PMN and enhances lung colonization through the generation of CAFs (Hoshino *et al*, 2015). Furthermore, lung‐resident CAFs are involved in establishing a PMN by secreting chemokines such as CXCL12 and CCL2. These chemokines can guide breast cancer, which express high levels of CXCR4 and CCR7, to the lung (Müller *et al*, 2001). IL1 and IL6 are two further cytokines that can activate CAFs through NF‐κB or JAK/STAT signaling, respectively (Erez *et al*, 2010; Sahai *et al*, 2020). CAFs trigger the NLRP3 inflammasome activation and secretion of pro‐inflammatory cytokines (such as IL1 and IL6), which results in a positive‐feedback loop generating evermore CAFs (Ershaid *et al*, 2019).

Tissue stiffness has a significant impact on the risk of breast cancer and the prognosis. A slight rise in tissue stiffness can activate YAP/TAZ mechano‐transducing pathways (Calvo *et al*, 2013; Lee *et al*, 2019). This leads to the transcription of different matricellular proteins and a more contractile phenotype of CAFs in general. Consequently, this aggravates tissue stiffness and results in a positive feedback‐loop that enhances CAF generation and increases oncogenic signaling in cancer cells (Panciera *et al*, 2020). Additionally, radiation‐ or chemotherapy may add to the physiological and genomic stress, thereby reinforcing this loop (Park *et al*, 2020). As shown by Wang *et al*, cerebral cavernous malformations 3 (CCM3) is a gatekeeper in this YAP‐mediated process and the loss of CCM3 in CAFs enhances tissue remodeling while exacerbating matrix stiffness. The result is reciprocal YAP/TAZ activation in neighboring tumor cells and dissemination to distant organs (Wang *et al*, 2021). There are also YAP‐independent mechano‐transducing processes that help breast cancer progression (Lee *et al*, 2019).

In brief, we have highlighted several CAF‐regulated FFLs that result in disease escalation. Various growth factors secreted from CAFs may facilitate the spread of cancer cells to other sites by remodeling the matrix and enhancing both angiogenesis and proliferation of cancer cells. Additionally, due to the secretion of pro‐inflammatory cytokines or exosomes that lead to an inflamed environment, the activity of CAFs may facilitate colonization of distant organs.

## Nutrients and metabolites support cancer cell survival at distant sites

### Metabolic activity during metastasis

Altered cellular metabolism is a hallmark of cancer and is as diverse as the disease itself (Hanahan & Weinberg, 2011). Metabolic pathways are intricate networks of highly plastic biochemical reactions that depend on many factors, including the presence of genetic mutations, enzymes, and nutrients (Elia *et al*, 2018). Nutrients converted to metabolites support various activities, including cellular homeostasis, growth, signaling, and epigenetic modifications (Elia *et al*, 2018).

Originally observed by Otto Warburg, tumors feature constitutive elevated glucose consumption and ATP generation through glycolysis, independent of the availability of oxygen (Warburg, 1956). This “aerobic glycolysis” was termed the “Warburg effect”. Nowadays, we know that while aerobic glycolysis does not yield the highest number of ATP per carbon atom, it is the source of crucial building blocks for several cellular metabolic pathways (Dey *et al*, 2021). Additionally, increased demand for NAD+ relative to ATP drives aerobic glycolysis (Luengo *et al*, 2021). However, tumor metabolism goes far beyond glycolysis. Another anabolic process active in cancer cells is oxidative phosphorylation (Davis *et al*, 2020). The metabolic activity of metastasizing breast cancer cells can vary according to subtype, for example, ER‐positive breast cancer cells are better adapted to oxidative phosphorylation and triple‐negative breast cancer cells to glycolysis (Lehúede *et al*, 2016).

Every organ is unique with regard to nutrients and oxygen availability, as well as to the level of oxidative stress (Schild *et al*, 2018). Tumor cells exhibit significant metabolic flexibility and, as a result, survive and thrive in different metastatic organs; the higher the metabolic flexibility, the higher the metastatic potential (Lehúede *et al*, 2016). Metabolic adaptation can occur on epigenetic and post‐translational levels through metabolite availability (Bergers & Fendt, 2021). Importantly, changes in metabolism affect cellular energetics and signaling networks (Schild *et al*, 2018). Thus, it is no surprise that many tumor mutations activate pro‐survival and growth pathways. For example, phosphatidylinositol 3‐kinase/protein kinase B (PI3K/AKT) and MYC are crucial proteins for cellular metabolism (Shim *et al*, 1997; Miricescu *et al*, 2021). In breast cancer, metabolic rewiring begins at the intravasation of cancer cells from the primary tumor and persists until colonization is complete (Elia *et al*, 2018; Lüönd *et al*, 2021). At these stages, nutrient availability and utilization dictate activity of metabolic pathways (Elia *et al*, 2017, 2018).

The metabolism of metastatic cancer cells is governed by the two overarching and intertwined concepts of metabolic plasticity and flexibility (Bergers & Fendt, 2021). Metabolic plasticity describes the involvement of specific metabolites in different pathways; prime examples are lactate, pyruvate, or glutamine. Metabolic flexibility on the other hand describes the use of different metabolites for one specific pathway.

In the following section, we summarize current knowledge about metabolic pathways and the nutritional cross‐feeding involved in breast cancer metastases. When nutrients are in short supply, changes in the microenvironment sustain the energetic state of cancer cells. For simplicity, we categorize the pathways as glycolytic, fatty acid, and amino acid metabolism.

### Nurturing the sugar high

Glucose is the most critical energy source for homeostatic tissues as well as for tumors (Sun *et al*, 2019; Santos & Hussain, 2020). Glycolysis‐related proteins such as glucose‐transporter 1 (GLUT‐1) and MCT4 are upregulated in patients’ breast cancer brain metastases (Kim *et al*, 2014). During glycolysis, glucose breaks down into two pyruvate molecules, and it has been shown that environmental pyruvate is involved in shaping the ECM in metastatic niches of the lung (Elia *et al*, 2019). Elevated pyruvate uptake results in increased α‐ketoglutarate‐enhancing collagen hydroxylation (Elia *et al*, 2019). Rapidly dividing cancer and myeloid cells consume most of the glucose within the TME (Reinfeld *et al*, 2021). Additionally, metabolic reprogramming in the TME toward aerobic glycolysis influences the immune system by attracting MDSCs via CCAAT/enhancer‐binding protein beta (CEBPB) and through G‐CSF and GM‐CSF secretion (Li *et al*, 2018).

**Figure 4:**
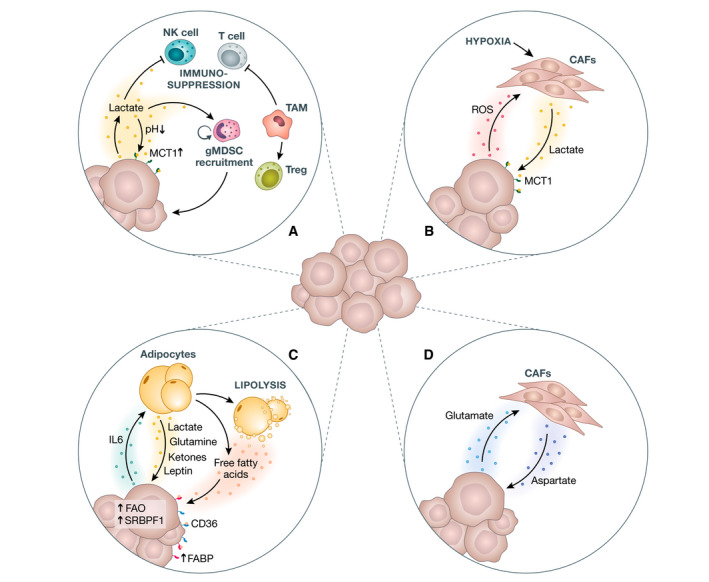
Nutritional feed‐forward loops fostering metastasis Factors secreted from cancer cells into the microenvironment generate an immune‐modulated and nutritionally beneficial niche. (A) Lactate from cancer cells lowers the extracellular pH. This is immunosuppressive to cytotoxic immune cells, including NK and T cells. Simultaneously, it attracts gMDSC to the metastatic niche, which further supports the growth of cancer. Additionally, uptake of the MCT1 transporter increases the availability of high‐energy substrate lactate. Moreover, MCT1 is upregulated in brain metastases. (B) ROS secreted from cancer cells together with a hypoxic microenvironment can stimulate lactate secretion from CAFs, which is then available via MCT1. (C) IL6 release promotes free fatty acids (FFA) secretion from adipocytes and also induces lipolysis in adjacent adipocytes, releasing even more FFAs into the TME. In turn, uptake of these FFAs into cancer cells via the CD36 lipid receptor or fatty acid‐binding proteins promotes further disease progression. Additionally, interaction of cancer cells and adipocytes stimulates release from the latter of lactate, glutamine, ketones, and leptin, which can enhance cancer cell growth. Elevated fatty acid oxidation (FAO) and upregulation of sterol regulatory element‐binding protein 1 (SREBP‐1) are both promotors of the metastatic cascade. (D) Example of nutritional cross‐feeding in the TME. Glutamate secreted from cancer cells promotes aspartate secretion from CAFs, which supports cancer cells metabolically. CAF, cancer‐associated fibroblast; FABP, fatty acid‐binding proteins; FAO, fatty acid oxidation; gMDSC, granulocytic myeloid‐derived suppressor cells; IL6, interleukin 6; MCT1, monocarboxylate transporter 1; ROS, reactive oxygen species.

Pyruvate generated by glucose or amino acids can be converted into lactate in a one‐step reaction catalyzed by lactate dehydrogenase (LDH) (Bergers & Fendt, 2021). Mainly produced by aerobic glycolytic cancer cells in hypoxic regions, lactate was long thought to be the ugly duckling of metabolites, being simply a waste product of glycolysis (Rabinowitz & Enerbäck, 2020). However, with the discovery that lactate can promote cancer cell growth, the spotlight turned on lactate and its activity as a pro‐tumorigenic metabolite (Fig 4A and B) (Boidot *et al*, 2012). Interestingly, hypoxia and cancer‐derived reactive oxygen species (ROS) can induce production and secretion of lactate by CAFs, thus promoting cancer cell metabolism (Fig 4B) and leading to a vicious FFL (Becker *et al*, 2020). Apart from being an energy‐rich metabolite enhancing cancer progression, lactate creates an acidic TME. This deters immune cell infiltration while simultaneously promoting pro‐tumorigenic immune cell populations, further exacerbating disease progression (Fig 4A) (Naik & Decock, 2020). Summarizing these findings, we highlight that glycolysis and its products pyruvate and lactate sustain breast cancer metastases.

### A fatty situation

Fatty acids (FAs) from de novo lipogenesis and exogenous uptake are linked to cancer progression (Elia *et al*, 2018). FA uptake occurs via lipid receptors CD36 and fatty acid‐binding proteins (FABPs). It has been shown that CD36 expression enhances the metastatic potential of DTCs in distant organs (Pascual *et al*, 2017). Concomitantly, the secretome of breast‐associated adipocytes induces FA uptake into breast cancer cells through CD36, enhancing primary tumor aggressiveness (Zaoui *et al*, 2019). Additionally, tumor cell activity can induce lipolysis in adjacent adipocytes. Free FAs then sustain the proliferation of cancer cells via FA oxidation, which is often upregulated in breast cancer cells (Fig 4C) (Wang *et al*, 2017; Broadfield *et al*, 2021). Thus, constant release of FA from adipocytes promotes cancer cell growth, which leads to even greater FA release from the TME (Romero *et al*, 2015).

De novo FA synthesis in cancer cells (based on glucose or glutamine) is the other main pathway of FA generation and this was shown recently to be involved in breast cancer brain metastasis (Ferraro *et al*, 2021). Additionally, upregulation of sterol regulatory‐binding protein 1 (SREBP1) in brain metastatic breast cancer cell lines promotes lipid synthesis and FA metabolism (Jin *et al*, 2020). Thus, SREBP‐1 promotes breast cancer metastases (Zhang *et al*, 2019). Notably, FAs are elevated in breast cancer metastases compared to the primary tumor, highlighting the importance of FA metabolism in metastatic disease progression (Liu *et al*, 2012).

### Amino acid addiction

Amino acids can act as an energy source, biosynthetic molecules, and mediators of redox balance in cancer cells, thus fueling anabolism of cancer cells and promoting metastasis.

Non‐essential amino acids (NEAAs) can be produced by host cells but are also available for cancer cells through dietary uptake. Proline catabolism is higher in metastases than in primary tumors of mice and human patients (Elia *et al*, 2017). Serine metabolism is important in breast cancer growth and progression (Mattaini *et al*, 2016). Indeed, the serine‐ and glycine‐limited brain environment normally restricts growth of metastatic seeds. However, phosphoglycerate dehydrogenase (PHGDH), which is important for the serine biosynthesis pathway, is upregulated in DTCs in the brain (Ngo *et al*, 2020). Ngo *et al* (2020) found that the low levels of serine and glycine render brain metastasis of MDA‐MB‐231 cells susceptible to PHGDH inhibition. PHGDH expression is involved in lung and liver metastasis and is linked to shorter overall survival in patients (Kim *et al*, 2014; Samanta *et al*, 2016; Rinaldi *et al*, 2021). For further reading on serine and one‐carbon metabolism in cancer, we refer to the review by Yang and Vousden (Yang & Vousden, 2016). Nutritional cross‐feeding was reported in stiff primary tumor niches, where CAFs receive glutamate from cancer cells and cancer cells receive aspartate from CAFs (Fig 4D) (Bertero *et al*, 2019). It is not known whether such a FFL also exists in breast cancer metastases.

Conditionally essential amino acids are only essential when the metabolic consumption exceeds the endogenous production. In cancer cells, these include arginine, asparagine, and glutamine (Bröer, 2020). Arginine metabolism transiently changes toward pro‐proliferative polyamine synthesis during metastasis of 4T1 cells in lungs (Kus *et al*, 2018). Increased glutamine metabolism is well described in breast cancer and glutamine is the most abundant free amino acid in the plasma (Cao *et al*, 2017; Bergers & Fendt, 2021). Immunotargeting of xCT, the glutamate and cysteine transporter, successfully inhibited breast cancer lung and brain metastases in preclinical mouse models (Lanzardo *et al*, 2016; Elia *et al*, 2018; Parida *et al*, 2022).

For breast cancer cells deprived of extracellular glutamine, asparagine becomes an essential amino acid, highlighting the interdependence of amino acids in the TME (Pavlova *et al*, 2018). Limiting asparagine bioavailability by dietary restriction reduces lung metastasis without affecting the primary tumor growth (Knott *et al*, 2018).

Amino acid exchange between cancer cells and their microenvironment supports metastasis (Bertero *et al*, 2019). However, there are few reports of nutritional cross‐feedings, as amino acids are primarily an anabolic need compared to secondary messengers modulating the microenvironment.

The different mechanisms of metabolic reprogramming described above promote metastasis and the survival of cancer cells under altered metabolic constraints (Bergers & Fendt, 2021). Homeostatic regulation of the tumor nutrient microenvironment in favor of metastasis offers unique opportunities to disrupt FFLs and specifically target metastasis formation.

## Outlook and clinical perspective

So far, a large proportion of cancer research and anti‐cancer drug development has focused on targeting the primary tumor. To extend therapeutic options for stage IV cancer patients, we need specifically targeted therapies aiming to eliminate metastasis. To accomplish such long‐term successful therapies, it is important to know the metastatic TME in detail, rather than using data based solely on primary tumors.

Technological advances are needed in order to understand in more detail the relationship between metastases and the TME, as well as the extent of intra‐ and inter‐metastatic and patient heterogeneity. Over the last decade, much progress has been made in characterizing tumors and their microenvironments at a single cell level as well as defining their spatial composition. Advanced technologies, such as single‐cell resolved imaging to distinguish intra‐tumoral heterogeneity with biosensors (i.e., for glycolysis), will provide powerful tools to unravel the metabolic states of metastatic cancer cells (Kondo *et al*, 2021). Similarly, new technologies such as “SpaceM”, which integrates light microscopy with MALDI‐imaging MS to spatially characterize in situ single‐cell metabolomics, are promising (Rappez *et al*, 2021). Additional new technologies include fluorouracil‐labeled RNA sequencing (Flura‐seq) of cancer cell RNAs (Basnet *et al*, 2019), which can characterize early colonization in different organs with high sensitivity *in situ* (Basnet *et al*, 2019). This technique has already been used, for example, to highlight specific metabolic requirements for DTCs growing in the lung microenvironment, as opposed to those growing in brain or breast (Basnet *et al*, 2019). Single‐cell metabolic profiling (scMEP) could help define metabolic characteristics of immune cell populations or cancer cells in metastases (Hartmann *et al*, 2021); which could be used to define how metabolism‐associated FFLs might be modulated and therapeutically targeted.

In this review, we provide some recent examples of how tumors and the microenvironment interact. However, one significant question persists: Is there a stage when the FFLs between the cancer and its TME are irreversible and the disease reaches a stage of no return? In the clinic, patients enter palliative care when this step of disease escalation is reached. If patients present with multiple metastatic lesions that are not resectable, targeting the cancer cells alone will be insufficient for disease eradication. Normalization of the microenvironment is crucial to improve therapeutic responses. Targeting FFLs may be a possible way to tackle cancer cell proliferation and normalize the environment and thus restore a homeostatic level in which cancer cells are suppressed by the microenvironment. However, targeting cells in the TME also poses the risk of enhanced side effects because cells apart from the metastatic niche have critical homeostatic activities. In this regard, it will be challenging to develop efficient therapies that target all sites at once, because the compositions of metastatic sites can be very different. It is tempting to speculate that the combination of immune‐related therapies and targeted cytotoxic agents will be more widely used to treat metastatic breast cancer. However, successful targeting of metastases will need a cautiously nuanced approach. Most importantly, cancer genomics and proteomics, tumor (sub)type, TME composition, and the metastatic organ need to be monitored with biopsies before and during therapy (Dey *et al*, 2021). Greater emphasis must be put on translationally relevant pre‐clinical studies that assess these factors in models of metastatic disease. Consequently, promising paths that should be investigated further are interventions that enhance anti‐tumor immune function while limiting cancer cell metabolism and proliferation (Dey *et al*, 2021). Intriguing perspectives are cancer vaccines and second generation immune‐checkpoint inhibitors.

Additionally, it should be noted that preclinical studies are often suboptimal compared to the clinical situation of patients. In‐depth preclinical research and clinical validation that focus on controlling dormant cells at the different metastatic sites are needed. The tumor dormancy window offers a means to prevent incipient metastases from reawakening. This has the advantage of intervening pharmacologically and/or by modifying risk factors at a stage when the number and heterogeneity of cancer cells are still relatively low. Moreover, technologies that identify harbingers of the switch from dormancy to metastatic outgrowth will allow therapeutic intervention during the early stages of metastases. These may include secreted cytokines and chemokines, as well as circulating cells, nucleotides, metabolites, and lipids. Nevertheless, given current detection methods, some patients present to the clinic only at later stages of disease when metastases are detectable, fully established and have already a rewired microenvironment; thus, research on late‐stage disease is also needed (Ganesh & Massagué, 2021). To summarize: with innovative ideas, more accurate model systems, access to patient material including on‐treatment biopsies, improved technologies, and combinations of targeted therapies with immunotherapies, researchers can pave the way to more effective treatment of metastatic patients.

## Author contributions

**Zora Baumann:** Conceptualization; Investigation; Visualization; Methodology; Writing—original draft; Writing—review and editing. **Priska Auf der Maur:** Conceptualization; Investigation; Visualization; Methodology; Writing—original draft; Writing—review and editing. **Mohamed Bentires‐Alj:** Supervision; Funding acquisition; Writing—original draft; Writing—review and editing.

In addition to the CRediT author contributions listed above, the contributions in detail are:

ZB, PADM, and MB‐A wrote and reviewed the manuscript. ZB and PADM designed the figures. ZB and PADM contributed equally, and order was chosen by flipping a coin. Both authors are allowed to refer to this article with their name in the first position.

## Disclosure and competing interests statement

ZB, PADM do not have any disclosures. MBA is a member of EMBO. MBA owns equities in, and receives laboratory support and compensation from Novartis, and serves as consultant for Basilea.

**Pending issues**

- Development of more sensitive biomarkers for premetastatic niches and minimal residual disease in patients.
- Therapies aiming at normalizing pre‐metastatic niches back to tumor suppressive homeostatic conditions.
- Design of drugs interrupting feed‐forward loops to halt disease progression efficiently.
- Development of treatments targeting specific metastatic sites.
- Investigating and defining points of no return in breast cancer disease progression to understand if they can be prevented.
- Improvement of translatability of preclinical to clinical studies.
- Creating pan‐cancer clinical trials targeting specific metastatic sites.
